# Bringing supply chain training opportunities closer to home—an experience with regional training institutes

**DOI:** 10.1186/2052-3211-7-S1-O15

**Published:** 2014-12-17

**Authors:** Motomoke Eomba, Kim Peacock, Rebecca Alban

**Affiliations:** 1 USAID | DELIVER PROJECT, John Snow Inc., Washington DC, USA

## Background

Since 1994, USAID and JSI have provided introductory supply chain courses for international audiences. As demand for these courses continued to grow, it became apparent that local delivery would increase the cost-effectiveness and sustainability of global SCM trainings. Starting in 2007, the USAID | DELIVER PROJECT built the capacity of four Regional Training Institutes (RTIs) that now offer high quality supply chain courses to an international audience in three languages (English, Spanish, and French).

## Method

The project developed detailed selection criteria to use when surveying and selecting RTIs, then trained and mentored the selected institutes in SCM, training/facilitation, marketing, and consulting. These capacity building interventions enabled the RTIs to design, package, price, market, and deliver capacity building programs; provide targeted technical assistance; and apply business savvy to their management and development activities.

## Results

Over time, the RTIs have evolved as leaders in training for SCM and logistics; they continue to offer high-quality training solutions in developing countries. Based in Peru, Tanzania, Burkina Faso, and South Africa, the RTIs leveraged local talent to provide training in commodity security and supply chain management of health commodities to the areas that needed it most. Today, the RTIs are either working entirely on their own, or with minimal technical assistance from the project. Financial support for trainings comes mainly from participants’ fees with diminishing support from USAID funds.

## Discussion

Project experience with the RTIs demonstrates that “facilitated outsourcing” of SCM trainings to regional training institutes can be a successful intervention to increase global supply chain training opportunities. While some objectives were met with quick success, others required significant technical assistance. RTIs have been successful in: recovering costs with their pricing structures, building consulting skills, forming a culture of entrepreneurship, and delivering high quality, highly rated courses. Challenges include: drafting adequate marketing plans to ensure sufficient enrolment, and ensuring ongoing quality control of courses.

## Lessons learned

Positive results include: reduced donor costs, creation of local opportunities for SCM professionals, and more participants trained each year. Specific attention must be given to: course pricing flexibility to meet market demands, formal evaluation/cost analysis of RTIs for evidence-building, and potential market saturation.

